# Tissue-Specific and Cation/Anion-Specific DNA Methylation Variations Occurred in *C. virgata* in Response to Salinity Stress

**DOI:** 10.1371/journal.pone.0078426

**Published:** 2013-11-05

**Authors:** Xiang Gao, Donghui Cao, Jie Liu, Xiaoping Wang, Shujuan Geng, Bao Liu, Decheng Shi

**Affiliations:** 1 Institutes of Genetics and Cytology, Northeast Normal University, Changchun, China; 2 Key Laboratory of Molecular Epigenetics of MOE, Institute of Genetics and Cytology, Northeast Normal University, Changchun, China; 3 Weifang University of science & technology, Shouguang, China; Rush University Medical Center, United States of America

## Abstract

Salinity is a widespread environmental problem limiting productivity and growth of plants. Halophytes which can adapt and resist certain salt stress have various mechanisms to defend the higher salinity and alkalinity, and epigenetic mechanisms especially DNA methylation may play important roles in plant adaptability and plasticity. In this study, we aimed to investigate the different influences of various single salts (NaCl, Na_2_SO_4_, NaHCO_3_, Na_2_CO_3_) and their mixed salts on halophyte *Chloris. virgata* from the DNA methylation prospective, and discover the underlying relationships between specific DNA methylation variations and specific cations/anions through the methylation-sensitive amplification polymorphism analysis. The results showed that the effects on DNA methylation variations of single salts were ranked as follows: Na_2_CO_3_> NaHCO_3_> Na_2_SO_4_> NaCl, and their mixed salts exerted tissue-specific effects on *C. virgata* seedlings. Eight types of DNA methylation variations were detected and defined in *C. virgata* according to the specific cations/anions existed in stressful solutions; in addition, mix-specific and higher pH-specific bands were the main type in leaves and roots independently. These findings suggested that mixed salts were not the simple combination of single salts. Furthermore, not only single salts but also mixed salts showed tissue-specific and cations/anions-specific DNA methylation variations.

## Introduction

In higher plants, DNA methylation mainly refers to 5’-methyl cytosine, and it presents in CG, CHG (H is any nucleotide) and CHH (H is A, T, C) sequences [1]. DNA methylation in plants is well known to be species-, tissue-, organelle- and age-specific [2], and is involved in the control of all genetic function including transcription, replication, DNA repair, gene transposition and cell differentiation [3]. Genome-wide high-resolution mapping and functional analysis of DNA methylation revealed that 8% of expressed genes were methylated in their promoters and 31% were in transcribed regions in rice [4].

Plants’ adaptability and plasticity in response to various environmental stresses are associated with the on-off status of the quantitative expressional level of key genes [5], and the on-off switch is mainly regulated by epigenetic status without changing the underlying nucleotide sequences in plant genome [6]. DNA methylation, as a conserved epigenetic regulation, is being studied extensively in recent years. DNA methylation persists through mitosis (short-term) and meiosis (long-term) to the next generation, which may contribute to the acquirement of resistance and adaptability of halophyte to salt stress [7], [8].

Salinity, a widespread and major environmental factor limiting plant growth and productivity, is known to exert changes on DNA methylation in a wild range of plants [9], [10], . Single salts stresses such as NaCl [13], NaHCO_3_ [14] and Na_2_CO_3_ [15] have been widely studied. On the other hand, combined salts, NaCl and Na_2_SO_4_, NaHCO_3_ and Na_2_CO_3_ have been defined and studied in our previous study [16]. The main causes of neutral salts (NaCl and Na_2_SO_4_) were ion toxicity and osmotic stress; besides that, another destructive cause in alkali salts (NaHCO_3_ and Na_2_CO_3_) was higher pH in the surrounding environment [17].

Although the previous studies implicated that alkali salts, with higher pH in solution, had more complex and destructive effects than neutral salts on DNA methylation, there are still more questions to be resolved. Firstly, which component in the stressful solution is the direct cause of the specific DNA methylation variation; furthermore, whether the influences of mixed salts are the combination of single salts or not; in addition, whether and how tissue-specific variations are present in leaves and roots of *C. virgata*. In this study, we attempted to understand the different influences of various single salts (NaCl, Na_2_SO_4_, NaHCO_3_, Na_2_CO_3_) and their mixed salts on DNA methylation, and to discover the underlying relationships between the stresses and DNA methylation. Better understanding of the regulation of DNA methylation in plant in response to specific cation or anion may create a new channel to cultivate new plants that can adapt to specific salt stresses.

## Materials and Methods

### Plant Materials and Stress Treatments

*C. virgata* seeds were collected from institutional native alkaline grassland located west of Jilin Province in China (44°40'–44°45' N, 123°44'–123°47' E). The seeds were sown in 17 cm diameter plastic pots (5 plants per pot) containing 2.5 kg of washed sand. After germination, the seedlings were watered with Hoagland nutrient solution once a day. All pots were placed in the greenhouse with the temperature at 25.0±1.5 °C during the day and 19.0±1.5°C at night; and photoperiod of 15/9 h (light/dark).

NaCl, Na_2_SO_4_, NaHCO_3_, Na_2_CO_3_ and their mixed salts (NaCl, Na_2_SO_4_, NaHCO_3_, Na_2_CO_3_, molar ratio 1∶1∶1∶1) were used to treat *C. virgata* seedlings at concentration of 200 mM, 150 mM, 200 mM, 50 mM and 200 mM respectively.

When the seedlings were 4 weeks old, only one seedling per pot was kept to make sure seedlings in all pots were growing uniformly. A total of 24 pots were selected and randomly divided into six sets. Each pot was considered as a single replicate; therefore there were four replicates per set. One set was used as a control (A), and the other five sets were used for NaCl (B), Na_2_SO_4_ (C), NaHCO_3_ (D), Na_2_CO_3_ (E), Mixed salts (F) treatment respectively. Stress treatments were performed once a day between 17:00–18:00 pm by watering plants thoroughly with nutrient solutions containing the corresponding salts. Control plants were watered with Hoagland nutrient solution only. The treatment lasted for 5 days.

### DNA Extraction and Methylation Sensitive Amplification Polymorphism (MSAP) Analysis

The leaves and roots of each individual *C. virgata* were rinsed gently with deionized water to remove sand and dried on filter paper, then ground to fine powder in liquid nitrogen. Genomic DNA was extracted following the modified cetyltrimethyl ammonium bromide (CTAB) method and purified by phenol extraction [18]. MSAP analysis was employed following the procedure of Vos et al. [19] using a pair of methylation sensitive isoschizomers, *Hpa*II or *Msp*I in combination with *EcoR*I. The *EcoR*I, H/M adapters, pre-amplification and selective amplification primers were listed in Table S1.

Considering that *Hpa*II is inactive when either of the cytosines in 5’-CCGG-3’ sites is fully methylated, whereas *Msp*I is sensitive only when the external cytosine is methylated. MSAP data originated from the PCR products can be converted into a binary matrix of 1 and 0 based on presence or absence of a certain band. If the band was present in *Hpa*II but absent in *Msp*I digestion, the pattern was marked as “H1M0”, indicating a methylation on the external cytosine (CHG methylation). In contrast, if the band was absent in *Hpa*II but present in *Msp*I digestion, the pattern was as “H0M1”, indicating that the internal cytosine was methylated (CG methylation). While pattern “H1M1” representing a non-methylated state, and pattern “H0M0” indicating either a external cytosine methylation or both cytosines were methylated. According to the methylation patterns that occurred in *C. virgata* seedlings under different salt treatments, ten different types of methylation changes were detected (Table S2).

### Statistical Analysis

Statistical analysis of MSAP results was performed as described by Cervera et al. [20]. Only clear and reproducible MSAP bands were scored. The levels of CG, CHG and the total methylation for a given plant sample were calculated. All data were represented as the average of the four replicates (independent plant individuals) and their standard errors (SE). The mean values were compared by Fishers least significant difference (LSD) test at p<0.05. Statistical analysis of the data was performed using the statistical software, SPSS 14.0 (SPSS, Chicago, IL, USA).

## Results

### Tissue-specific DNA methylation in *C. virgata* in response to different salt treatments

Leaves and roots of *C. virgata* were used to assess DNA methylation levels under four single salts (B-E sets) and their mixed salts (F set) treatments. After PCR amplification using 26 pairs of selective primers, 966 and 962 bands were detected in leaves and roots respectively in control *C. virgata* (A set), and their methylation levels (MASP %) were 18.29% and 13.31% respectively, with CG methylation (15.73% in leaves and 11.33% in roots) being the main type (Table 1). These results showed that the average methylation level in *C. virgata* leaves was higher than in roots; and the higher methylation level was due to the higher CG methylation level.

**Table 1:** Effects of NaCl, Na_2_SO_4_, NaHCO_3_, Na_2_CO_3_, Mix salt stresses on cytosine methylation in *C.virgata* leaves and roots.

Organ	Treatment	Total amplified bands	Non-methylated CCGG sites		Methylated CCGG sites				Total methylated bands^c^	MSAP (%)^d^
			Sites	Ratios (%)	Full methyl^a^		Hemimethyl^b^			
					CG sites	Ratio (%)	CHG sites	Ratio (%)		
Leaf	Control	966	789	81.68	152	15.73	25	2.56	177	18.29
	NaCl	967	791	81.80	151	15.62	25	2.56	176	18.17
	Na_2_SO_4_	968	799	82.54	143	14.77	26	2.66	169	17.43
	NaHCO_3_	968	802	82.85	140	14.46	26	2.66	166	17.12
	Na_2_CO_3_	969	805	83.08	138	14.24	26	2.66	164	16.90
	Mix	952	808	84.87	121	12.71	23	2.35	144	15.06
Root	Control	962	834	86.69	109	11.33	19	1.97	128	13.31
	NaCl	984	862	87.60	105	10.67	17	1.73	122	12.40
	Na_2_SO_4_	985	855	86.80	110	11.17	20	2.03	130	13.20
	NaHCO_3_	995	859	86.33	110	11.06	26	2.61	136	13.67
	Na_2_CO_3_	979	845	86.31	110	11.24	24	2.45	134	13.69
	Mix	975	847	86.87	104	10.67	24	2.46	128	13.13

a Full methyl (ation) represents those digested by EcoRI/MspI but not by EcoRI/HpaII.

b Hemimethyl (ation) represents those digested by EcoRI/HpaII but not by EcoRI/MspI.

c Total methylated bands = a/b.

d MSAP (%)  = c/ Total amplified bands*100.

**Figure 1:**
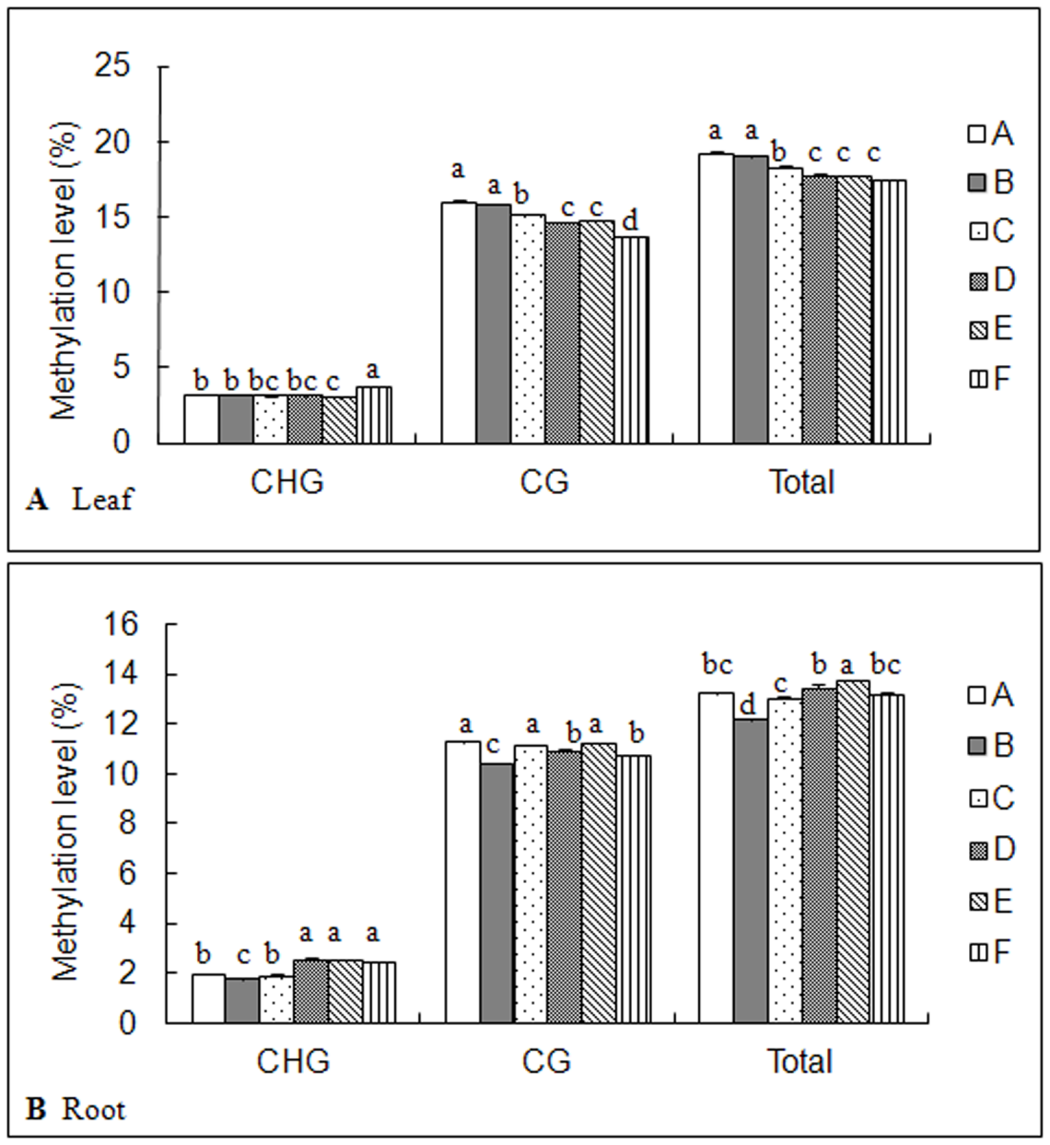
Effects of different salt stresses on cytosine methylation level in *C.virgata*. A: control, B: NaCl, C: Na_2_SO_4_, D: NaHCO_3_, E: Na_2_CO_3_, F: Mix. Data represent means ±S.E. of four replicates. Different lower-case letters represent significant difference among treatments at the 5% level, according to least significant difference (LSD) test.

Comparing with control set, all treatments (B-F sets) led to changes of DNA methylation levels in both leaves and roots. In *C. virgata* leaves, the total methylation levels remained largely unchanged in B set, but was decreased dramatically in C-F sets (p<0.05) (Figure 1a). More complicated influences were observed in *C. virgata* roots, the total methylation level was decreased in B set but increased in E set dramatically (p<0.05) (Figure 1b), while remained largely unchanged in the other sets. We noted that mixed salts treatment decreased the methylation level (p<0.05) in leaves but not in roots.

### DNA methylation variation in *C. virgata* seedlings under different salt treatments

**Figure 2:**
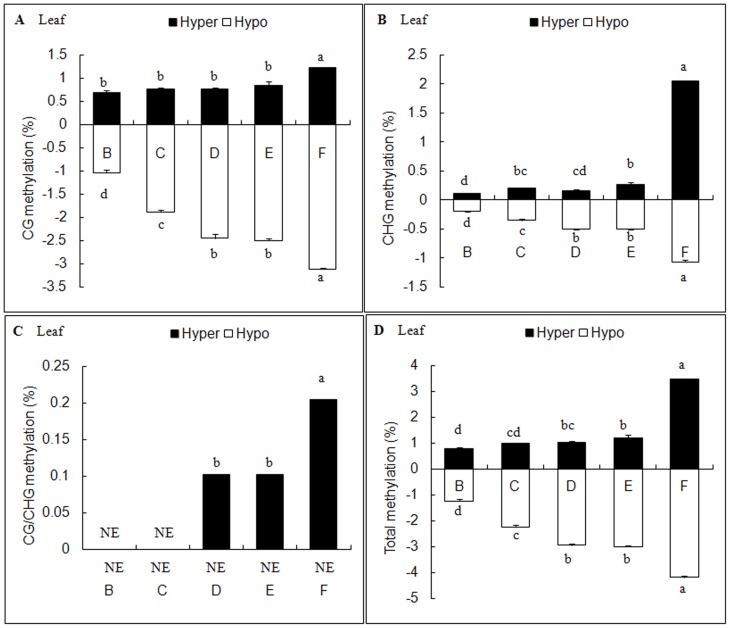
Effects of different salt stresses on methylation variation in *C.virgata* leaves. B: NaCl, C: Na_2_SO_4_, D: NaHCO_3_, E: Na_2_CO_3_, F: Mix. Data represent means ±S.E. of four replicates. Different lower-case letters represent significant difference among treatments at the 5% level, according to least significant difference (LSD) test. NE: Not exist.

**Figure 3:**
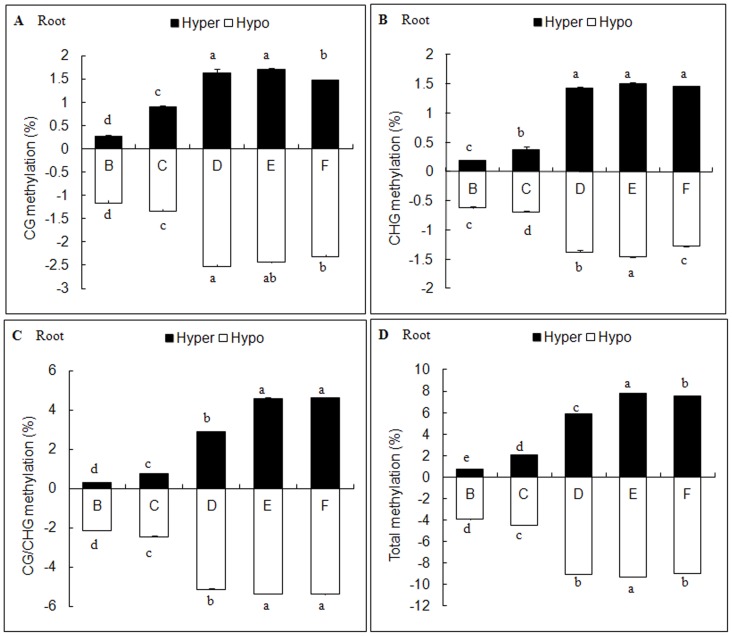
Effects of different salt stresses on methylation variation in *C.virgata* roots. B: NaCl, C: Na_2_SO_4_, D: NaHCO_3_, E: Na_2_CO_3_, F: Mix. Data represent means ±S.E. of four replicates. Different lower-case letters represent significant difference among treatments at the 5% level, according to least significant difference (LSD) test.

According to the methylation patterns under different salt treatments, ten types of DNA methylation variations were detected (Table S2). All salt treatments (B-F) caused DNA methylation variations including hyper-methylation and hypo-methylation in leaves and roots; however, the extents of variation occurred discriminately (Figure 2, 3). We also found that the main variation type was hypo-methylation in both leaves and roots.

**Figure 4:**
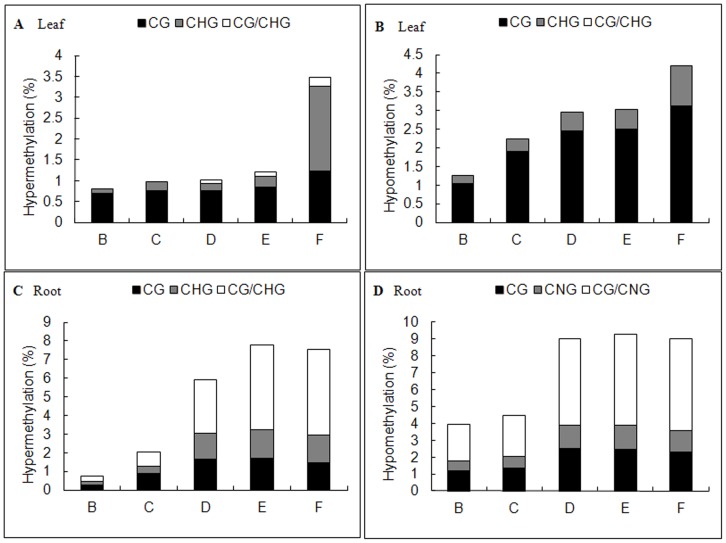
Effects of different salt stresses on hypermethylation (a, c) and hypomethylation (b, d) in *C.virgata*. B: NaCl, C: Na_2_SO_4_, D: NaHCO_3_, E: Na_2_CO_3_, F: Mix.

In *C. virgata* leaves, the total variation frequencies that occurred in B-F sets were 2.04%, 3.22%, 3.99%, 4.22%, 7.67% respectively (Figure 2d), while in roots were 4.69%, 7.53%, 14.96%, 17.08%, 16.55% respectively (Figure 3d). Comparing with E set, the mixed salt treatment (F set) still increased the variation frequency in leaves (p<0.05) but decreased in roots. The results also indicated that the variation frequencies were higher in alkali salt treatments (D and E sets) than neutral salt treatments (B and C sets) in both leaves (Figure 4a, b) and roots (Figure 4c, d). Furthermore, based on their effects on DNA methylation variation frequencies, the salts were ranked as follows: Na_2_CO_3_>NaHCO_3_>Na_2_SO_4_>NaCl. In addition, mixed salts caused tissue-specific DNA methylation variations; it strengthened the effect of each single salt in leaves but weaken the effect in roots. Besides that, DNA methylation variations were influenced much more seriously in roots than in leaves under each of stress treatments.

### The cation/anion-specific DNA methylation variations occurred in *C. virgata* seedlings under different salt treatments

All single salt treatments (B-E sets) had Na^+^ and one specific anion, and the mixed salt treatment (F set) had Na^+^ and all the anions. Besides that, alkali salt treatments (D and E sets) had higher-pH factor. According to their specificity to different salt treatments, variations can be classified into eight regular types: Na^+^; Cl^−^-; SO_4_^2−^-; HCO_3_^−^-; CO_3_^2−^-; Mix-(the bands changed specially in F set); higher pH- (the bands changed specially in the salt treatments with higher-pH) (D-F sets); and lower pH-specific (the bands changed specially in the salt treatments with lower-pH) (B and C sets). We also found other non-specific bands but were not analyzed in our research.

To distinguish the bands clearly, schema charts showing hyper-methylation and hypo-methylation were made according to the MSAP electrophoresis results. We found that in *C. virgata* leaves, of the 28 hyper-methylated and 30 hypo-methylated bands, the main type was Mix-specific band, accounting for 20 (71.43%) and 13 (43.33%) respectively. In contrast, in *C. virgata* roots, of the 66 hyper-methylated and 89 hypo-methylated bands, the main type was higher-pH specific bands, accounting for 43 (65.15%) and 57 (64.04%) respectively. The results showed that cation/anion specific variations existed in different salt treatments; in addition, the main methylation variation type was hypo-methylation both in roots and leaves. Our conclusion that more DNA methylation variations occurred in roots than in leaves was consistent with the SPSS analysis result.

## Discussion

In recent years, evidence is growing in favor of DNA methylation participating in the adaptability of environmental stresses through regulation of gene expression networks [8]. Demethylation leading to transcriptional activation of certain functionally inactive genes [21], and interphase chromatin structural changes in rDNA loci [22] were detected in higher plants exposed to abiotic stresses. Hypermethylation of higher plants in response to abiotic stresses was mainly present in heterochromatic loci [9]. DNA hypermethylation and demethylation were also detected in halophyte *C. virgata* under neutral and alkaline salt treatment in our previous study [16], but the definite cause that led to DNA methylation variations have not been discovered. Since salinity factors existed in soil are complicated, better understanding of specific cause-effect relationship between DNA methylation variations and cations/anions surrounding the plants is essential for cultivating new plants to adapt complex salinity stresses.

Tissue-specific gene expression is known to be controlled by DNA methylation. In this research, tissue-specific DNA methylation levels were detected in *C. virgata* leaves and roots (Table 1), which were consistent with previous studies [23], [24], [25]. Furthermore, a lower level was observed in roots, indicating unique biological functions of *C. virgata* leaves and roots in response to salt stresses. In addition, different salt stresses exerted tissue-specific variations, decreasing the total methylation level in leaves (p<0.05) but had no consistent effects in roots (Figure 1). While consistent with previous reports on tissue-dependent DNA methylation pattern and its possible function in regulating tissue-specific gene expression [12], the results suggested that tissue specificity of epigenetic changes might be an essential regulatory mechanism for halophyte *C. virgata* to resist and adapt adverse environments.

**Figure 5:**
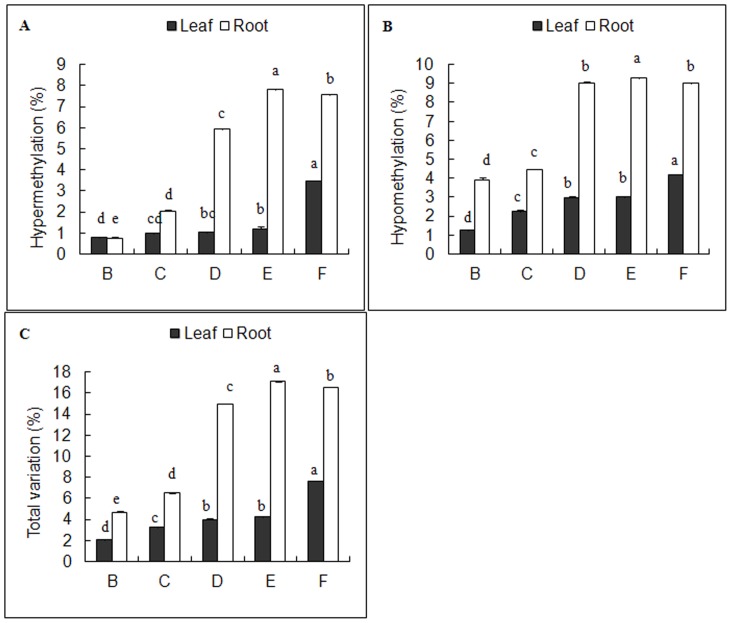
Effects of different salt stresses on variations in *C.virgata* leaves and roots. B: NaCl, C: Na_2_SO_4_, D: NaHCO_3_, E: Na_2_CO_3_, F: Mix. Data represent means ±S.E. of four replicates. Different lower-case letters represent significant difference among treatments at the 5% level, according to least significant difference (LSD) test.

**Figure 6:**
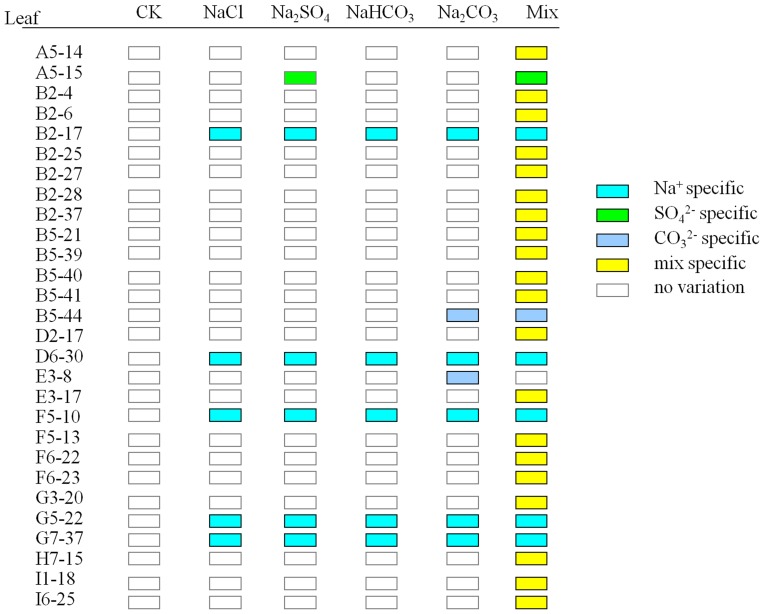
Specific hypermethylation bands occurred in *C.virgata* leaves. The hollow rectangles indicated the bands not changed, while solid rectangles indicated the hyper-methylated bands. “Capital letter +Arabic number” indicated the primer combination of HM, and the Arabic numbers indicated the order of variations bands amplified in this primer combination.

**Figure 7:**
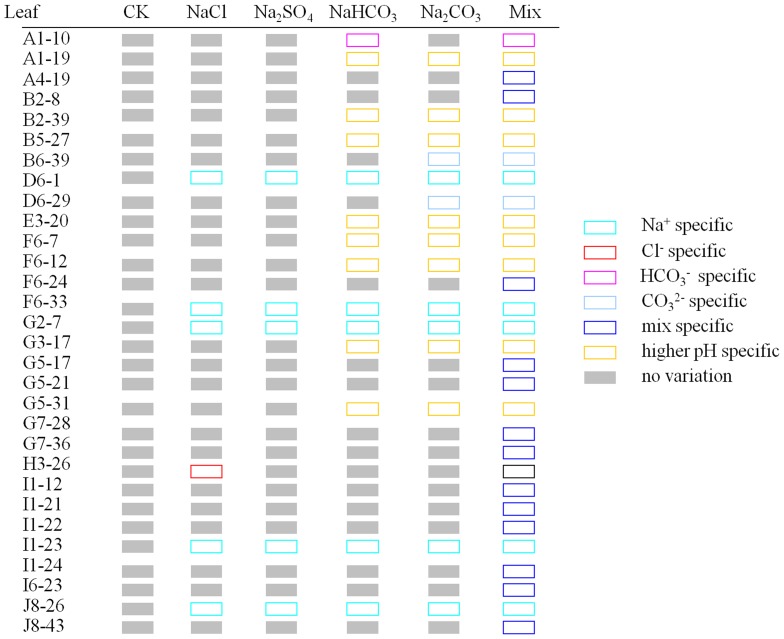
Specific hypomethylation bands occurred in *C.virgata* leaves. The solid rectangles indicated the bands not changed, while hollow rectangles indicated the hypo-methylated bands. “Capital letter +Arabic number” indicated the primer combination of HM, and the arabic numbers indicated the order of variations bands amplified in this primer combination.

**Figure 8:**
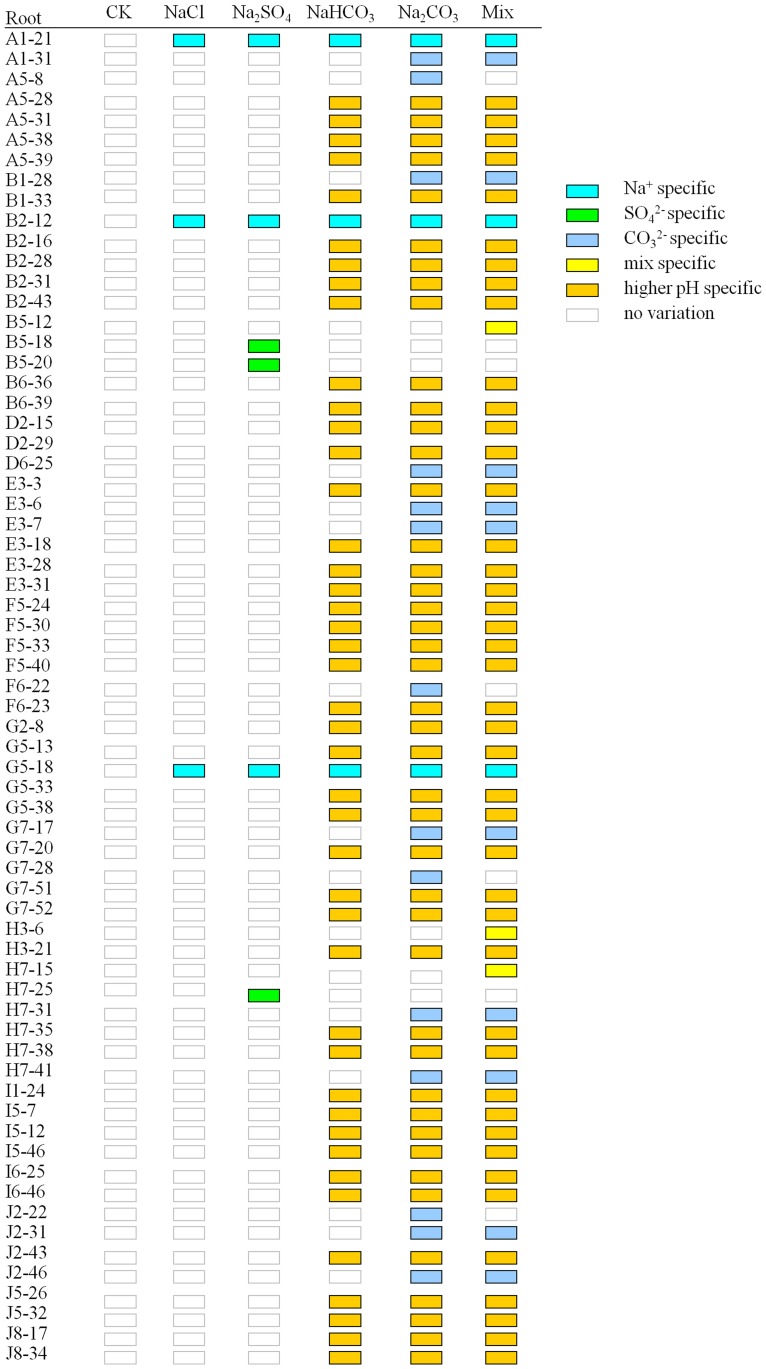
Specific hypermethylation bands occurred in *C.virgata* roots. The hollow rectangles indicated the bands not changed, while solid rectangles indicated the hyper-methylated bands. “Capital letter +Arabic number” indicated the primer combination of HM, and the Arabic numbers indicated the order of variations bands amplified in this primer combination.

**Figure 9:**
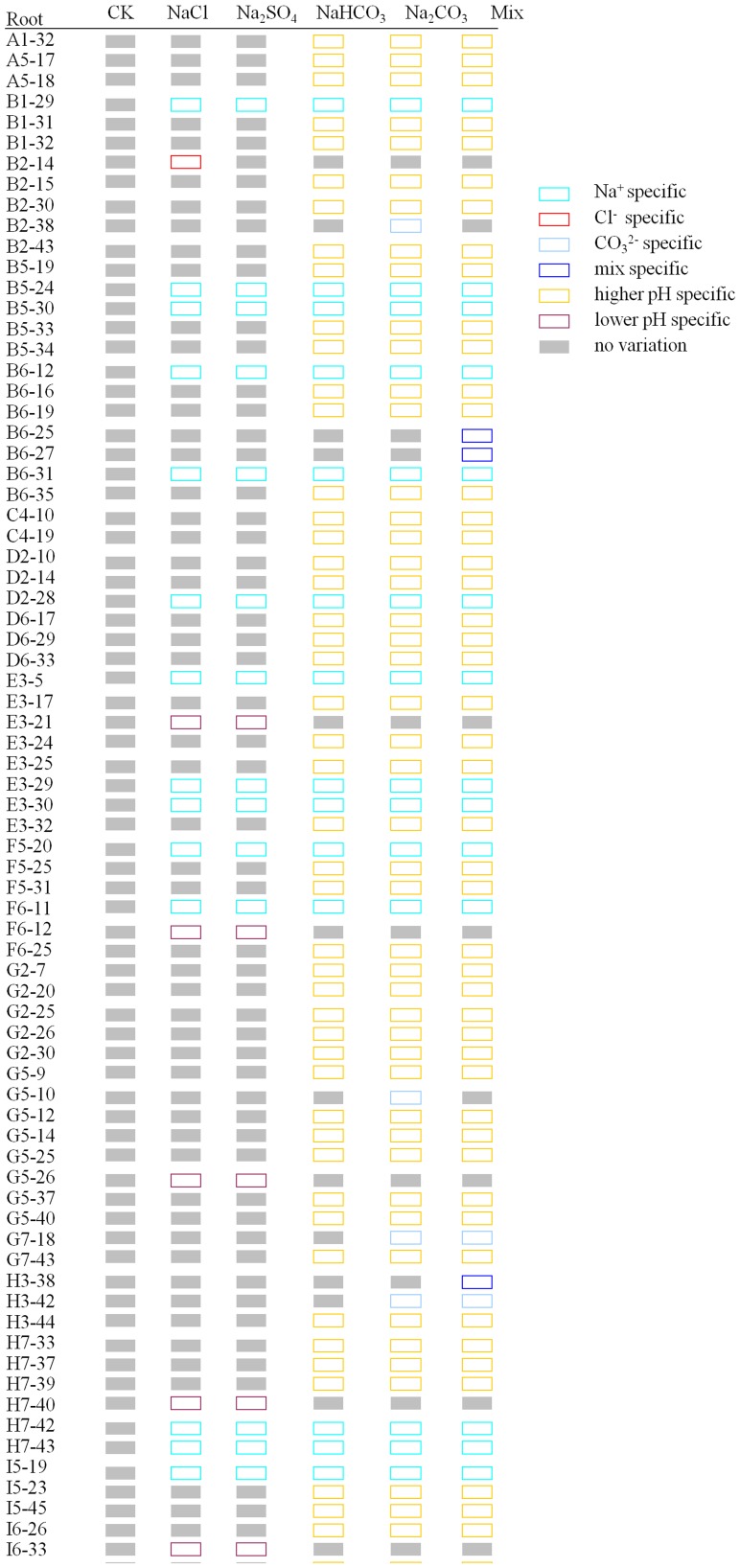
Specific hypomethylation bands occurred in *C.virgata* roots. The solid rectangles indicated the bands not changed, while hollow rectangles indicated the hypo-methylated bands. “Capital letter +Arabic number” indicated the primer combination of HM, and the Arabic numbers indicated the order of variations bands amplified in this primer combination.

Alkali salts (NaHCO_3_ and Na_2_CO_3_), with a higherpH, had more destructive effects than neutral salts (NaCl and Na_2_SO_4_) with a neutral pH (Figure 3, 4), and their effects were ranked as follows: Na_2_CO_3_> NaHCO_3_> Na_2_SO_4_> NaCl (Figure 5). The toxicity of neutral salts to plants mainly comes from ion damage and osmotic stress, while higher pH is another lethal factor limiting plant growth and development in alkali salts [26]. However, when the four kinds of single salt were mixed together, mixed salts (F set) enhanced the DNA methylation variation in leaves but weakened it in roots (Figure 5). These results suggested that the mixed salts were not the simple combination of single salt but had more complicated effects, as further supported by the Mix-specific hyper or hypo-methylation bands identified in F set (Figure 6–9). The interaction among Na^+^, Cl^−^, SO_4_^2−^, HCO_3_^−^ and CO_3_^2−^ in controlling methylation might be one of mechanisms that certain plants could be alive in higher concentration mixed salts but sensitive to lower single salt.

Mix-specific bands represented the biggest proportion of the eight types of variation bands in *C. virgata* leaves. However, the main type in roots was higher-pH specific bands (Figure 6–9). It was possible that the tissue specific methylation variations were due to the fact that roots directly contacted, experienced and reacted with higher-pH in the surrounding environment. Several organic acids were secreted from *C. virgata* root, and they were demonstrated to be key components to adjust higher pH level in the peripheral environment [27].

Hypomethylation occurred more frequently in both *C. virgata* leaves and roots (Figure 2, 3) in our research. It suggested that more stress responsive genes were activated in response to salinity stress. The reduction of methylation levels in maize gene *ZmMI1* [28] and tobacco gene *NtGPDL* [29] induced by abiotic stresses were detected. Hypomethylation was reported to affect plant development [30], and hypomethylation at transposable elements (TEs) induced by abiotic stress trigged 21nt siRNA biogenesis which served as a conduit for transgenerational memory [31]. The initial studies had confirmed that epigenetic states and certain environmental responses in seed plants could persist in the next sexual generation [32]. Enhanced stress tolerance encoded in DNA methylation information was conferred upon the progeny [33]. It suggested that acclimation and accumulation of salinity resistance and tolerance of halophyte *C. virgata* through DNA methylation variation inheritance to next generation made *C. virgata* to be a pioneer plant growing in saline and alkaline soils.

In the evolution procedure of higher plants surviving from various environmental stresses, the key factors that are implicated in epigenetic memory are not limited to DNA methylation, post-translational histone modification and small RNA molecules are largely responsible for regulating the transcriptional genome output [34]. Tissue-specific and cation/anion-specific DNA methylation variations were detected in our research; future analysis of the global histone and miRNA modification will allow comprehensive understanding of the epigenetic mechanisms in response to abiotic and biotic stresses.

## Supporting Information

Table S1 Sequences of adapters, pre-amplification primers, and selective amplification primers in MSAP analysis.
(DOCX)

Table S2 The changes in methylation patterns occurred in *C. virgata* seedlings under different salt treatments. H indicates *Eco*RI/*Hpa*II digestion; M indicates *EcoR*I/*Msp*I digestion.
(DOCX)
