# Supplementary material for: Tissue-Specific and Cation/Anion-Specific DNA Methylation Variations Occurred in C. virgata in Response to Salinity Stress
Source: PLoS One. 2013 Nov 5;8(11):e78426. doi: 10.1371/journal.pone.0078426 (PMC3818329; doi:10.1371/journal.pone.0078426)
Supplement: Table S1 — Sequences of adapters, pre-amplification primers, and selective amplification primers in MSAP analysis. (DOCX) [file pone.0078426.s001.docx]

**Table S1.** Sequences of adapters, pre-amplification primers and selective amplification primers in MSAP analysis.

|  | *Eco*RI (E) | *Hpa*II/*Msp*I (H/M) |
| --- | --- | --- |
| Adapter-1 | CTCGTAGACTGCGTACC | GATCATGAGTCCTGCT |
| Adapter-2 | AATTGGTACGCAGTC | CGAGCAGGACTCATGA |
| Preamplication primers | GACTGCGTACCAATTCA | ATCATGAGTCCTGCTGGT |
| Selective amplication primers | A: GACTGCGTACCAATTCAAC | 1: ATCATGAGTCCTGCTCGG TCT |
|  | B: GACTGCGTACCAATTCAAG | 2: ATCATGAGTCCTGCTCGG TCG |
|  | C: GACTGCGTACCAATTCACA | 3: ATCATGAGTCCTGCTCGG TCC |
|  | D: GACTGCGTACCAATTCACT | 4: ATCATGAGTCCTGCTCGG TTC |
|  | E: GACTGCGTACCAATTCACC | 5: ATCATGAGTCCTGCTCGG TTG |
|  | F: GACTGCGTACCAATTCACG | 6: ATCATGAGTCCTGCTCGG TTA |
|  | G: GACTGCGTACCAATTCAGC | 7: ATCATGAGTCCTGCTCGG TGA |
|  | H: GACTGCGTACCAATTCAGG | 8: ATCATGAGTCCTGCTCGG TGT |
|  | I: GACTGCGTACCAATTCAGA | 9: ATCATGAGTCCTGCTCGG TGC |
|  | J: GACTGCGTACCAATTCATC | 10: ATCATGAGTCCTGCTCGG TAC |
