# Supplementary material for: Tissue-Specific and Cation/Anion-Specific DNA Methylation Variations Occurred in C. virgata in Response to Salinity Stress
Source: PLoS One. 2013 Nov 5;8(11):e78426. doi: 10.1371/journal.pone.0078426 (PMC3818329; doi:10.1371/journal.pone.0078426)
Supplement: Table S2 — The changes in methylation patterns occurred in C. virgata seedlings under different salt treatments. H indicates EcoRI/HpaII digestion; M indicates EcoRI/MspI digestion. (DOCX) [file pone.0078426.s002.docx]

**Table S2.** The changes in methylation patterns occurred in *C. virgata* seedlings under different salt treatments. H indicates *Eco*RI/*Hpa*II digestion; M indicates *EcoR*I/*Msp*I digestion.

|  | Class | Changes in methylation | Digestion products | | | |
| --- | --- | --- | --- | --- | --- | --- |
|  |  |  | Control | | Treated | |
|  |  |  | H | M | H | M |
| Methylation Events | M1 | CG Hyper | 1 | 1 | 0 | 1 |
|  | M2 | CHG Hyper | 1 | 1 | 1 | 0 |
|  | M3 | CG/CHG Hyper | 1 | 1 | 0 | 0 |
|  | M4 | CHG Hyper | 0 | 1 | 0 | 0 |
|  | M5 | CG Hyper | 1 | 0 | 0 | 0 |
| Demethylation Events | D1 | CG Hypo | 0 | 1 | 1 | 1 |
|  | D2 | CHG Hypo | 1 | 0 | 1 | 1 |
|  | D3 | CG/CHG Hypo | 0 | 0 | 1 | 1 |
|  | D4 | CHG Hypo | 0 | 0 | 0 | 1 |
|  | D5 | CG Hypo | 0 | 0 | 1 | 0 |
